# OpenBiodiv-O: ontology of the OpenBiodiv knowledge management system

**DOI:** 10.1186/s13326-017-0174-5

**Published:** 2018-01-18

**Authors:** Viktor Senderov, Kiril Simov, Nico Franz, Pavel Stoev, Terry Catapano, Donat Agosti, Guido Sautter, Robert A. Morris, Lyubomir Penev

**Affiliations:** 1 Pensoft Publishers, Prof. Georgi Zlatarski 12, Sofia, 1700 Bulgaria; 2 Institute of Biodiversity and Ecosystems Research, Bulgarian Academy of Sciences, Sofia, Bulgaria; 3 Institute of Information and Communication Technologies, Bulgarian Academy of Sciences, Sofia, Bulgaria; 4 Arizona State University, School of Life Sciences, Tempe Campus, Tempe, 4501 AZ USA; 5 Plazi, Bern, Switzerland; 6 University of Massachusetts at Boston, Boston, USA; 7 National Museum of Natural History, 1 Tsar Osvoboditel Blvd., Sofia, 1000 Bulgaria

**Keywords:** Biodiversity, Biodiversity informatics, Semantic web, Semantic publishing, Ontology, Knowledge management, Linked open data, RDF, OWL, Taxonomy, Concept taxonomy, Biological systematics, Data modeling

## Abstract

**Background:**

The biodiversity domain, and in particular biological taxonomy, is moving in the direction of semantization of its research outputs. The present work introduces OpenBiodiv-O, the ontology that serves as the basis of the OpenBiodiv Knowledge Management System. Our intent is to provide an ontology that fills the gaps between ontologies for biodiversity resources, such as DarwinCore-based ontologies, and semantic publishing ontologies, such as the SPAR Ontologies. We bridge this gap by providing an ontology focusing on biological taxonomy.

**Results:**

OpenBiodiv-O introduces classes, properties, and axioms in the domains of scholarly biodiversity publishing and biological taxonomy and aligns them with several important domain ontologies (FaBiO, DoCO, DwC, Darwin-SW, NOMEN, ENVO). By doing so, it bridges the ontological gap across scholarly biodiversity publishing and biological taxonomy and allows for the creation of a Linked Open Dataset (LOD) of biodiversity information (a biodiversity knowledge graph) and enables the creation of the OpenBiodiv Knowledge Management System.

A key feature of the ontology is that it is an ontology of the scientific process of biological taxonomy and not of any particular state of knowledge. This feature allows it to express a multiplicity of scientific opinions. The resulting OpenBiodiv knowledge system may gain a high level of trust in the scientific community as it does not force a scientific opinion on its users (e.g. practicing taxonomists, library researchers, etc.), but rather provides the tools for experts to encode different views as science progresses.

**Conclusions:**

OpenBiodiv-O provides a conceptual model of the structure of a biodiversity publication and the development of related taxonomic concepts. It also serves as the basis for the OpenBiodiv Knowledge Management System.

**Electronic supplementary material:**

The online version of this article (doi:10.1186/s13326-017-0174-5) contains supplementary material, which is available to authorized users.

## Background

The desire for an integrated information system serving the needs of the biodiversity community dates at least as far back as 1985 when the Taxonomy Database Working Group (TDWG)—later renamed to Biodiversity Informatics Standards—was established [1]. In 1999, the Global Biodiversity Information Facility (GBIF) was created after the Organization for Economic Cooperation and Development (OECD) had arrived at the conclusion that “an international mechanism is needed to make biodiversity data and information accessible worldwide” [2]. The Bouchout declaration [3] crowned the results of the pro-iBiosphere project (2012 - 2014) [4] dedicated to the task of creating an integrated biodiversity information system. The Bouchout declaration proposes to make scholarly biodiversity knowledge freely available as Linked Open Data. A parallel process in the U.S.A. started even earlier with the establishment of the Global Names Architecture [5, 6].

The specification and design of a semantic system, the Open Biodiversity Knowledge Management System (OBKMS, later simply OpenBiodiv), implementing the objectives of the Bouchout Declaration by focusing on knowledge extraction from academic journals and research databases, were outlined amongst others in [7, 8]. In this publication we present the OpenBiodiv Ontology (OpenBiodiv-O)—the knowledge and inferencing model of OpenBiodiv [9]. OpenBiodiv-O provides a conceptual model of the structure of a biodiversity publication and the development of related taxonomic concepts.

### Previous work

In the biomedical domain there are well-established efforts to extract information and discover knowledge from literature [10–12]. The biodiversity domain, and in particular biological systematics and taxonomy (from here on in this paper referred to as *taxonomy*), is also moving in the direction of semantization of its research outputs [13–15]. The publishing domain has been modeled through the Semantic Publishing and Referencing Ontologies (SPAR Ontologies) [16]. The SPAR Ontologies are a collection of ontologies incorporating—amongst others—FaBiO, the FRBR-aligned Bibliographic Ontology [17], and DoCO, the Document Component Ontology [18]. The SPAR Ontologies provide a set of classes and properties for the description of general-purpose journal articles, their components, and related publishing resources. Taxonomic articles and their components, on the other hand, have been modeled through the TaxPub XML Document Type Definition (DTD) (also referred to loosely as XML schema) and the Treatment Ontologies [19, 20]. While TaxPub is the XML-schema of taxonomic publishing for several important taxonomic journals (e.g. ZooKeys, Biodiversity Data Journal), the Treatment Ontologies are still in development and have served as a conceptual template for OpenBiodiv-O. In fact, they share many of the same authors.

Taxonomic nomenclature is a discipline with a very long tradition. It transitioned to its modern form with the publication of the Linnaean System [21]. Already by the beginning of the last century, there were hundreds of vocabulary terms (e.g. *types*) [22]. At present the naming of organismal groups is governed by by the International Code of Zoological Nomenclature (ICZN) [23] and by the International Code of Nomenclature for algae, fungi, and plants (Melbourne Code) [24]. Due to their complexity (e.g. ICZN has 18 chapters and 3 appendices), it proved challenging to create a top-down ontology of biological nomenclature. Example attempts include the relatively complete NOMEN ontology [25] and the somewhat less complete Taxonomic Nomenclatural Status Terms (TNSS) [26].

There are several projects that are aimed at modeling the broader biodiversity domain conceptually. Darwin Semantic Web (Darwin-SW) [27] adapts the previously existing Darwin Core (DwC) terms [28] as RDF. These models deal primarily with organismal occurrence data.

Modeling and formalization of the strictly taxonomic domain has been discussed by Berendsohn [29] and later, e.g., in [30, 31]. Noteworthy efforts are the XML-based Taxonomic Concept Transfer Schema [32] and a now defunct Taxon Concept ontology [33].

### Aims

The present work introduces OpenBiodiv-O, which serves as the basis of OpenBiodiv. By developing an ontology focusing on biological taxonomy, our intent is to provide an ontology that fills in the gaps between ontologies for biodiversity resources such as Darwin-SW and semantic publishing ontologies such as the ontologies comprising the SPAR Ontologies. Moreover, we take the view that it is advantageous to model the taxonomic process itself rather than any particular state of knowledge.

OpenBiodiv [8] “lifts” biodiversity information from scholarly publications and academic databases into a computable semantic form. The implementation of the system will be treated in future works. In this contribution, we discuss OpenBiodiv-O by first introducing the modeled domain conceptually and then formalizing it in “Results” section.

### Domain description

Biological taxonomy is a very old discipline dating back possibly to Aristotle, whose fundamental insight was to group living things in a hierarchy [34]. The discipline took its modern form after Carl Linnaeus (1707 - 1778) [34]. In his *Systema Naturae* Linnaeus proposed to group organisms into *kingdoms, classes, orders, genera*, and *species* bearing latinized scientific names with a strictly prescribed syntax. Linnaeus listed possible alternative names and gave a characteristic description of the groups [21]. These groups are called *taxa*, which is a Greek word for *arrangement*. The hierarchy that taxa form is called taxonomy. The etymology of the word is Greek and roughly translates to *method of arranging*. Note the polysemy here: the science of biological taxonomy is called taxonomy as is the arrangement of taxa itself. We believe, however, that it is sufficiently clear from context what is meant by “taxonomy” in any particular usage throughout this paper.

Even though Linnaeus and his colleagues may have hoped to describe life on Earth during their lifetimes, we now know that there are millions of species still undiscovered and undescribed [35]. On the other hand, our understanding of species and higher-rank taxonomic concepts changes as evolutionary biology advances [36]. Therefore, an accurate and evolutionarily reliable description of life on Earth is a perpetual process and cannot be completed with a single project that can be converted into an ontology. Thus, our aim is not to create an ontology capturing a fixed view of biological taxonomy, but to create an ontology of the taxonomic process. The ongoing use of this ontology will enable the formal description of taxonomic biodiversity knowledge at any given point in time. In the following paragraphs, we introduce what the taxonomic process entails and reflect on the resources that need modeling.

An examination of the taxonomic process reveals that taxonomy works by employing the scientific method: researchers examine specimens and, based on the phenotypic and genetic variation that they observe, form a hypothesis [37]. This hypothesis may be called a taxonomic concept, a potential taxon, a species hypothesis [29], or an operational taxonomic unit (OTU) [38] in the case of a numerically delimited taxon.

A taxonomic concept describes the allowable phenotypic, genomic, or other variation within a taxon by designating type specimens and describing characters explicitly. It is a valid falsifiable scientific claim as it needs to fulfill certain verifiable evolutionary requirements. For example, a species-rank taxonomic hypothesis needs to fit our current understanding of species (species concept, [36]). More generally, the aspiration is that species concepts are adequate and give certain tangible criteria for species delimitation. However, valid scientific discussions continue about concept adequacy. The discussions are nuanced because they often draw on different conceptions of the relative weight of certain evolutionary phenomena. This leads to having quite a few different species concepts—morphological, ecological, phylogenetic, genomic, biological, etc. [36]. Nevertheless, if we fix a species concept—let us say we take the biological species concept—we can falsify any given species-rank taxonomic hypothesis against our fixed species concept.

Similarly, hypotheses of higher rank (representing upper levels of the taxonomic hierarchy) also need to fulfill certain evolutionary requirements. For example, a modern genus concept requires all species assigned to it to be descendants of a separate lineage and to form a monophyletic clade.

The ranks (taxonomy hierarchy levels) are not completely fixed. The usage of lower ranks (species, genus, family, order) is governed by international Codes [23, 24]. In the example of Linnaeus’ ranks, each organism is first a member of its species, then genus, then order, then class, and finally kingdom. Which specific ranks a given taxonomic study employs is dependent on the field (e.g. botany vs. zoology), on the particular author, on the level of taxonomic resolution required, as well as on the history of classifying in that particular group.

Once the researchers have formed their concept, it must be published in a scientific outlet (journal or book). The biological Codes put some requirements and recommendations aimed at ensuring the quality of published research but ultimately it is a democratic process guaranteeing that everyone may publish taxonomic concepts provided they follow the rules of the Codes. This means that in order to create a knowledge base of biodiversity, we need to be able to mine taxonomic papers from legacy and modern journals and books.

As a first good approximation, a taxonomic concept is based on a number of specimens or occurrences that are listed in a section usually called “Materials Examined”. In general terms, we can say that a sighting of a living thing, i.e. an organism, at a given location and at a given time is referred to as an occurrence, and a voucher for this occurrence (e.g. the sampling of the organism itself) is referred to as a specimen [27]. Moreover, a taxonomic article may include other specialized sections such as the Checklist section, where one may list all taxa (in fact: the taxonomic concepts for those taxa) for organisms observed in a given region.

Typically, the information content of a treatment consists of several units. First, we have the aforementioned nomenclatural information that pertains to the scientific name—its authorship, etymology, related names, etc. Then, we have the taxonomic concept information that can be considered to have two components, as well: the first one is the intensional component of the taxonomic concept made up mostly of *traits* or *characters*. Traits are an explicit definition of the allowable variation (e.g. phenotypic, genomic, or ecological) of the organisms that make up the taxon. For example, we can define the order of spiders, Araneae, to be the class of organisms that have specialized appendages used for sperm transfer called pedipalps [39]. Knowledge of this kind is found in the Diagnosis, Description, Distribution and other subsections of the treatment.

Non-traditionally delimited taxonomic hypotheses are called *operational taxonomic units* (OTU’s). In the case of genomic delimitation, sometimes the concepts are published directly as database entries and not as Code-compliant taxonomic articles [40]. A genomic delimitation can, for example, be based on a barcode sequence and on a statistical clustering algorithm specifying the allowable sequence variability that an organism can possess in order to be considered part of the barcode sequence-bearing operational taxonomic unit. However, as, in the general case, we don’t have a Linnaean name or a morphological description for an operational taxonomic unit, we refer to it as a *dark taxon* [40]. The term “dark” is, however, usually reserved for concepts at lower ranks. Operational taxonomic units are published, for example, in the form of *barcode identification numbers* (BIN’s) in the Barcode of Life Data Systems (BOLD) [41], or as *species hypotheses* in Unified system for the DNA based fungal species linked to the classification (UNITE) [42].

The second part of the information content of a taxonomic concept is the ostensive component: a listing of some (but not necessarily all) of the organisms that belong to the taxonomic concept. This information is found in the Materials Examined subsection of the treatment.

Finally, the relationships between taxonomic concepts—simple hierarchical (*is a*) or more fine-grained Region Connection Calculus 5 (RCC-5) [30, 43]—can be both intensionally defined in the nomenclature section or ostensively inferred from the Materials Examined. However, given the customary idiosyncrasies of biological descriptions, providing an initial set of RCC-5 relationships for a machine reasoner to work with often requires expert assessment and cannot be easily lifted from the text.

Thus, in order to model the taxonomic process, our ontology models scholarly taxonomic papers, database entries, agents responsible for their creation, treatments, taxonomic concepts, scientific names, occurrence and specimen information, other entities (e.g. ecological, geographical) part-taking in the taxonomic process, as well as relationships among these.

## Methods

OpenBiodiv-O is expressed in Resource Description Framework (RDF). At the onset of the project [8], a consideration was made to use RDF in favor of a more complex data model such as Neo4J’s. The choice of RDF was made in order to be able to incorporate the multitude of existing domain ontologies into the overall model.

To develop the conceptualization of the taxonomic process and then the ontology we utilized the following process: (1) domain analysis and identification of important resources and their relationships; (2) analysis of existing data models and ontologies and identification of missing classes and properties for the successful formalization of the domain.

The formal structure of the ontology is specified by employing the RDF Schema (RDFS) and the Web Ontology Language (OWL). It is encoded as a part of a literate programming [44] document titled “OpenBiodiv Ontology and Guide” [45]. The structure has been extracted from that file via *knitr* and provided here as Additional file 1. It is also possible to request the ontology via Curl from the endpoint with the indication of content-type: application/rdf+xml. The vocabularies can be found as more additional files: Taxonomic Statuses (Additional file 2) and RCC-5 (Additional file 3), on the website [9], and on the GitHub page [46] (under ontology/).

A partial dataset from Pensoft’s journals has been generated with OpenBiodiv-O and can be found at the SPARQL Endpoint <http://graph.openbiodiv.net/>, select repository obkms_i6. The endpoint is also accessible from the website, <http://openbiodiv.net/>, under “SPARQL Endpoint”. Demos are available as “Saved Queries” from the workbench.

## Results

We understand OpenBiodiv-O to be the *shared formal specification of the conceptualization* [47–49] that we have introduced in Background. OpenBiodiv-O describes the structure of this conceptualization, not any particular state of it.

There are several domains in which the modeled resources fall. The first one is the scholarly biodiversity publishing domain. The second domain is that of taxonomic nomenclature. The third domain is that of broader taxonomic (biodiversity) resources (e.g. taxonomic concepts and their relationships, species occurrences, traits). To combine such disparate resources together we rely on SKOS [50]. Unless otherwise noted, the default namespace of the classes and properties for this paper is <http://openbiodiv.net/>. The prefixes discussed in this paper are listed in Additional file 1, at the beginning of the ontology.

### Semantic modeling of the biodiversity publishing domain

An article as such may be represented by a set of metadata, while its content consists of article components such as sections, tables, figures and so on [51].

To accommodate the specific needs of scholarly biodiversity publishing, we introduce a new class for taxonomic articles, Taxonomic Article (:TaxonomicArticle), new classes for specific subsections of the taxonomic article such as Taxonomic Treatment, Taxonomic Key, and Taxonomic Checklist, and a new class, Taxonomic Name Usage (:TaxonomicNameUsage), for the mentioning of a taxonomic name (see next subsection) in an article. These new classes are summarized in Table 1.

**Table 1:** New biodiversity publishing classes introduced

Class QName	Comment
:Treatment	Section of a taxonomic article
:NomenclatureSection	Subsection of Treatment
:NomenclatureHeading	Contains a nomenclatural act
:NomenclatureCitationList	List of citations of related concepts
:MaterialsExamined	List of examined specimens
:BiologySection	Subsection of Treatment
:DescriptionSection	Subsection of Treatment
:TaxonomicKey	Section with an identification key
:TaxonomicChecklist	Section with a list of taxa for a region
:TaxonomicNameUsage	Mention of a taxonomic name

The classes from this subsection are based on the TaxPub XML Document Type Definition (DTD) [19] (also referred to loosely as XML schema), on the structure of Biodiversity Data Journal’s taxonomic paper [52], and and on the Treatment Ontologies [20].

Furthermore, we introduce two properties: *contains* (:contains) and *mentions* (:mentions). *Contains* is used to link parts of the article together and *mentions* links parts of the article to other concepts.

**Fig. 1:**
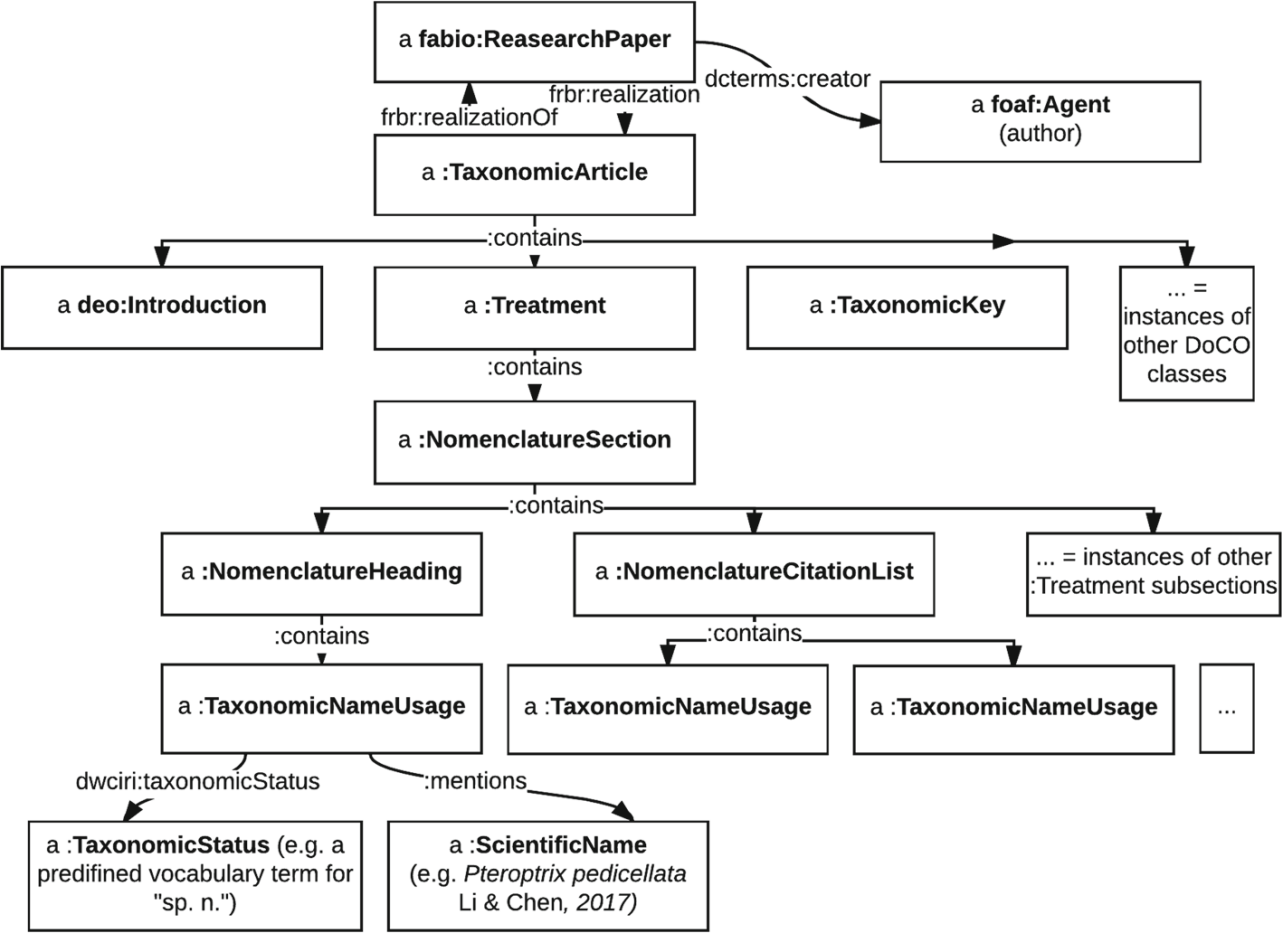
Taxonomic article diagram. A graphical representation of the relationships between instances of the publishing-related classes that OpenBiodiv introduces

A graphical representation of the relationships between instances of the publishing-related classes that OpenBiodiv introduces is to be found in the diagram in Fig. 1.

#### Semantics, alignment, and usage

Our bibliographic model has the Semantic Publishing and Referencing Ontologies (SPAR Ontologies) at its core with a few extensions that we have written to accommodate for taxonomic elements. The SPAR Ontologies’ FRBR-aligned Bibliographic Ontology (FaBiO) uses the Functional Requirements for Bibliographic Records (FRBR) [53] model to separate publishable items into less or more abstract classes. We deal primarily with the Work class, i.e. the conceptual idea behind a publishable item (e.g. the story of “War and Peace” as thought up by Leo Tolstoy), and the Expression class, i.e. a version of record of a Work (e.g. “War and Peace,” paperback edition by Wordsworth Classics).

Taxonomic Article is a subclass of FaBiO’s Journal Article. Furthermore Journal Article is a FRBR Expression. This implies that taxonomic articles are FRBR expressions as well. This has important implications later on when discussing taxonomic concept labels. Also, it means that we separate the abstract properties of an article (in a FaBiO Research Paper instance, which is a Work) from the version of record (in a Taxonomic Article, an Expression).

The taxonomic-specific section and subsection classes are introduced as subclasses of Discourse Element Ontology’s (DEO) Discourse Element (deo:DiscourseElement, [18]). So is the class Mention (:Mention), meant to represent an area of a document that can be considered a mention of something. This class, and the corresponding property, *mentions*, are inspired by pext:Mention and its corresponding property from PROTON [54]. The redefinition is necessary by the fact in OpenBiodiv-O they possess a slightly different semantics and a different placement in the upper-level hierarchy. We then introduce Taxonomic Name Usage as a subclass of Mention.

**Fig. 2:**
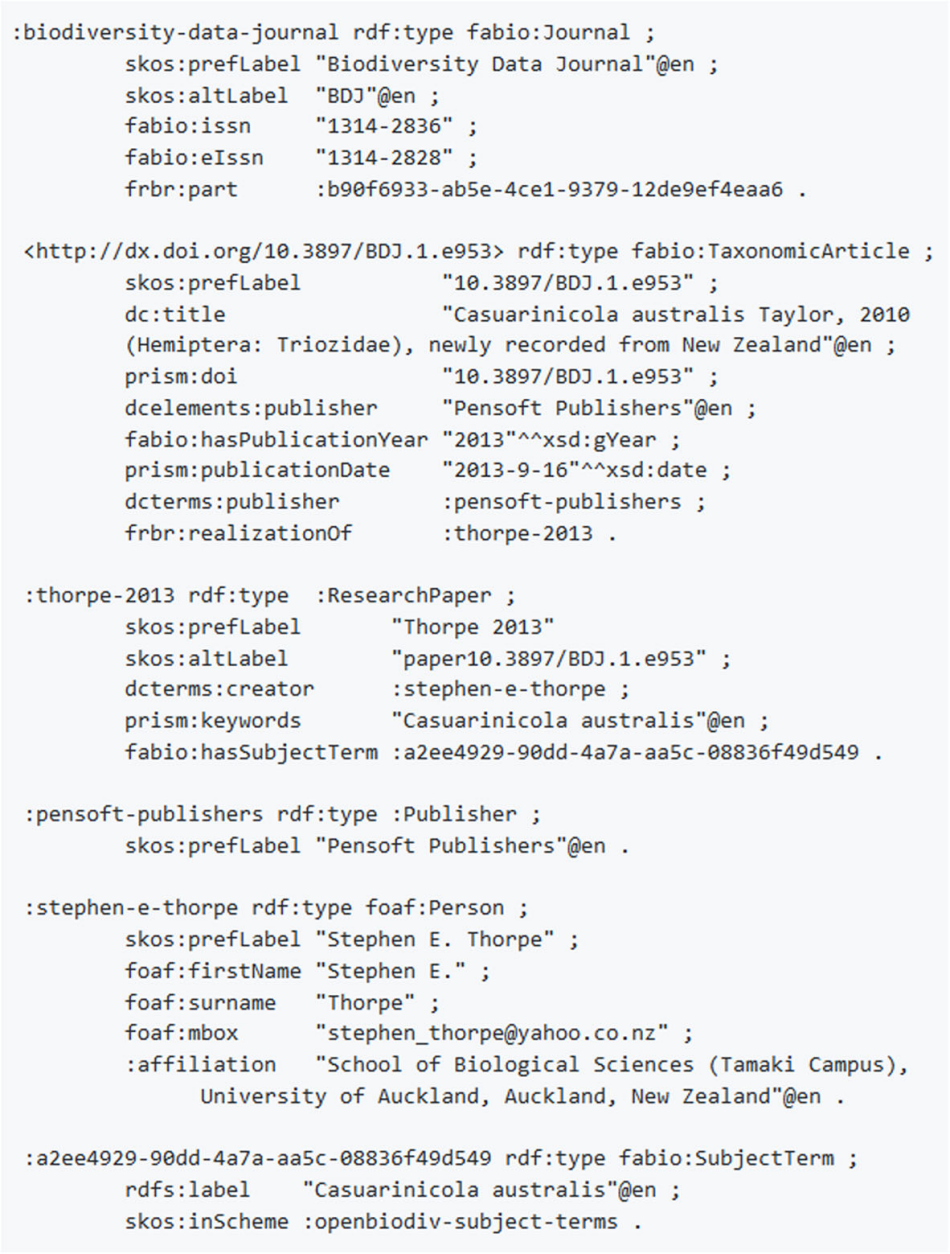
Example article metadata. This example shows how to express the metadata of a taxonomic article with the SPAR Ontologies’ model and the classes that OpenBiodiv defines. The code is in Turtle

**Fig. 3:**
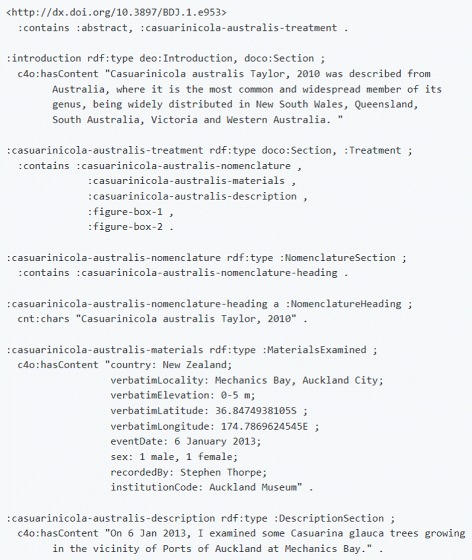
Example article structure. This examples shows how to express the article structure with the help of :contains. The code is in Turtle

This placement of the document component classes that we’ve introduced in Discourse Element means that they ought to be used exactly in the same way as one would use the other discourse elements from DEO and DoCO (analogous to e.g. deo:Introduction). Note: DEO is imported by DoCO. Figures 2 and 3 give example usage in Turtle illustrating these ideas. A caveat here is that while the SPAR Ontologies use po:contains in their examples, we use *contains*, which is a subproperty of po:contains with the additional property of being transitive. We believe this definition is sensible as surely a sub-subcomponent is contained in a component. All other aspects of expressing a taxonomic article in RDF according to OpenBiodiv-O are exactly the same as according to the SPAR Ontologies.

### Semantic modeling of biological nomenclature

While NOMEN and TNSS (introduced in subsection “Previous work”) take a top-down approach of modeling the nomenclatural Codes, OpenBiodiv-O takes a bottom-up approach of modeling the use of taxonomic names in articles. Where possible we align OpenBiodiv-O classes to NOMEN.

**Fig. 4:**
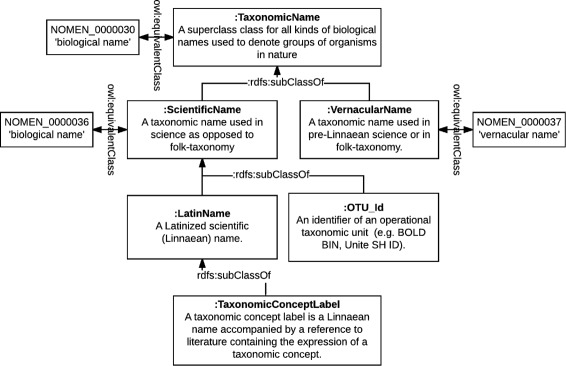
Taxonomic name class hierarchy diagram. We created this class hierarchy to accommodate both traditional taxonomic name usages and the usage of taxonomic concept labels and operational taxonomic units

Based on the need to accommodate taxonomic concepts, we have defined the class hierarchy of taxonomic names found in Fig. 4. Furthermore, we have introduced the class Taxonomic Name Usage (:TaxonomicNameUsage). Taxonomic name usages have been discussed widely in the community (e.g. in [55]); however, the meaning of term remains vague. The abbreviation TNU is used interchangeably for “taxon name usage” and for “taxonomic name usage.” In OpenBiodiv-O, a taxonomic name usage is the mentioning of a taxonomic name in the text, optionally followed by a taxonomic status.

For example, “*Heser stoevi* Deltschev 2016, sp. n.” is a taxonomic name usage. The cursive text followed by the author and year of the original species description is the latinized scientific name. The abbreviation “sp. n.” stands for the Latin *species novum*, indicating the discovery of a new taxon.

We also introduce the class Taxonomic Concept Label (:TaxonomicConceptLabel). A taxonomic concept label (TCL) is a Linnaean name plus a reference to a publication, where the discussed taxon is circumscribed. The link is via the keyword “sec.” (Latin for *secundum*) [29]. An example would be "*Andropogon virginicus* var. *tenuispatheus* sec. Blomquist (1948)". Here, Blomquist (1948) is a reference to [56], the publication where the concept is circumscribed.

We extracted taxonomic status abbreviations from about 4000 articles across four taxonomic journals (ZooKeys, Biodiversity Data Journal, PhytoKeys, and MycoKeys) in order to create a taxonomic status vocabulary (Additional file 2) that covers the eight most common cases (Table 2). The Latin abbreviations that have been classified into these classes can be found on the OpenBiodiv GitHub page [46] (See “Methods” section for more details).

**Table 2:** OpenBiodiv taxonomic status vocabulary

Vocabulary instance QName	Example abbrev	Comment
:TaxonomicUncertainty	*incertae sedis*	Taxonomic uncertainty
:TaxonDiscovery	*sp. n.*	Taxonomic discovery
:ReplacementName	*comb. n.*	Replacement name
:UnavailableName	*nomen dubium*	Unavailable name
:AvailableName	*stat. rev.*	Available name
:TypeSpecimenDesignation	*lectotype designation*	Type specimen designation
:TypeSpeciesDesignation	*type species*	Type species designation
:NewOccurrenceRecord	*new country record*	New occurrence record (for region)

**Fig. 5:**
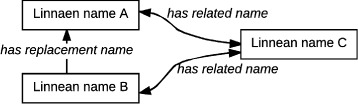
Scientific name patterns diagram. Chains of *replacement names* can be followed to find the currently used name. *Related name* indicates that two names are related somehow, but not which one is preferable

Based on our analysis of taxonomic statuses, we have identified two Code-compliant patterns of relationship between latinized scientific names (Fig. 5). The pattern *replacement name*, implemented via the property :replacementName, indicates that a certain Linnaean name should be used instead of another Linnaean name. It covers a wide variety of cases in the Codes, such as, for example, the placement of one species taxon in a new genus (“comb. n.”), the correction of a name for nomenclatural reasons (“nomen novum”), or the application of the Principle of Priority for the discovery of synonyms (“syn. nov.”) [23].

The other pattern is that of *related names* (:relatedName). It is a broader pattern, indicating that two names are somehow related. For example, they may be synonyms, with one replacing the other, or they may point to taxonomically related taxonomic concepts. For example, *Harmonia manillana* (Mulsant, 1866) is related to *Caria manillana* Mulsant 1866 since, as per [57], a name-bearing type (lectotype) of *Harmonia manillana* (Mulsant, 1866) sec. Poorani [57] is named *Caria manillana* Mulsant 1866.

#### Semantics, alignment and usage

As evident from Fig. 4, OpenBiodiv-O taxonomic names are aligned to NOMEN names.

**Fig. 6:**
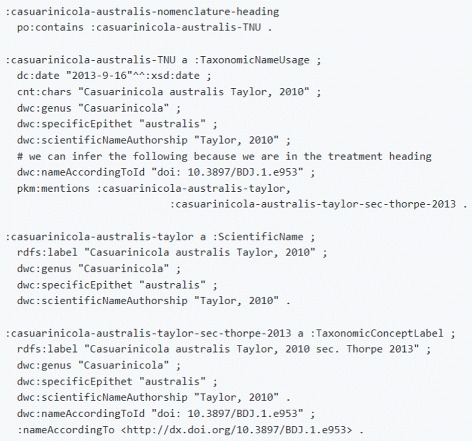
Example taxonomic name usage. This examples shows how taxonomic name usages link document components to taxonomic names. The code is in Turtle

The linking between text and taxonomic names must pass through the intermediary class Taxonomic Name Usage. As parts of the manuscript, taxonomic name usages link document components to taxonomic names. Taxonomic name usages are *contained* in sections such as Treatment, and *mention* a taxonomic name as illustrated in the example in Fig. 6.

### Semantic modeling of the taxonomic concepts

**Fig. 7:**
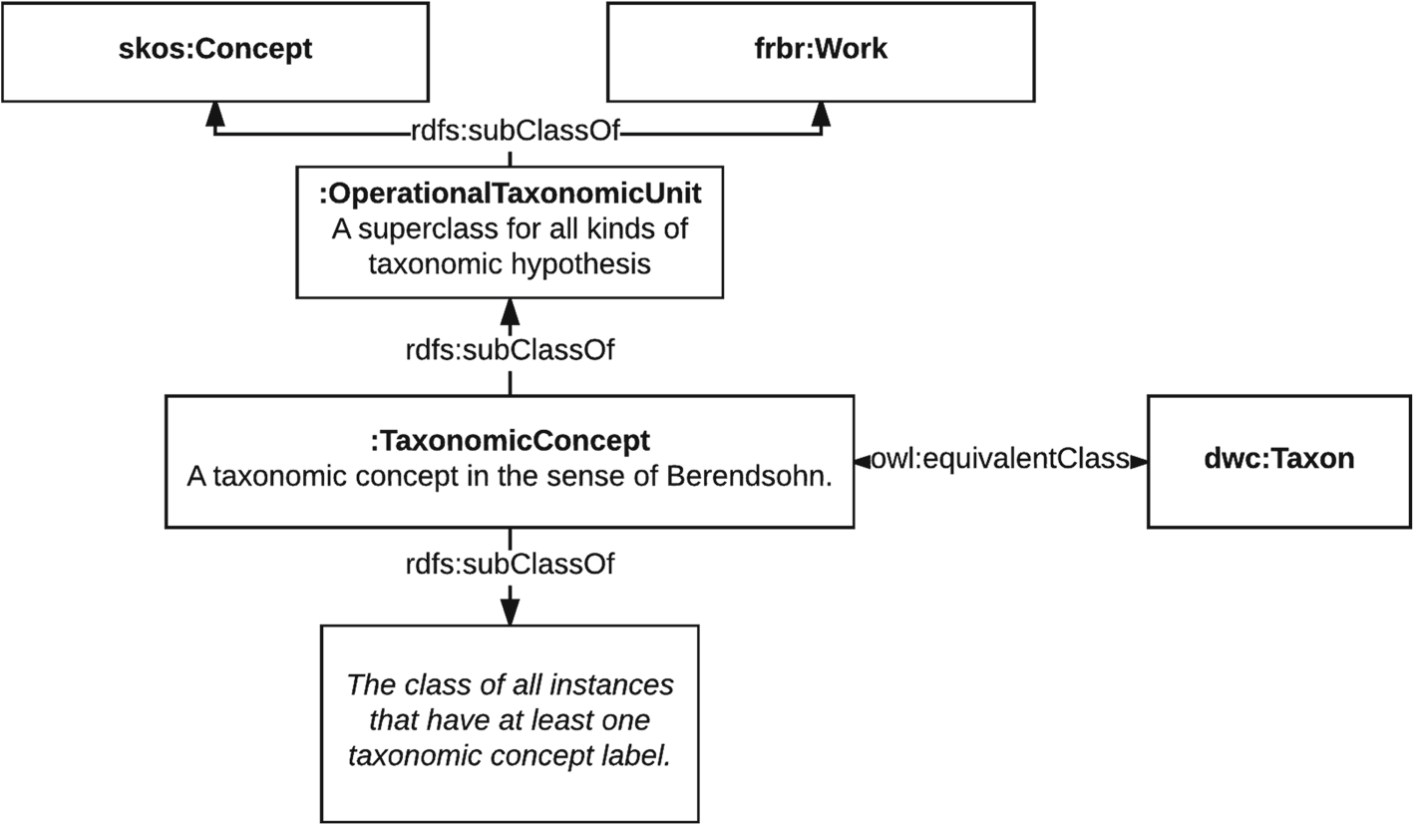
Taxonomic concept diagram. A taxonomic concept is a skos:Concept, a frbr:Work, a dwc:Taxon and has at least one taxonomic concept label

In OpenBiodiv-O taxonomic names are not the carriers of semantic information about taxa. This task is accomplished by a new class, Taxonomic Concept (:TaxonomicConcept). A taxonomic concept is the theory that a taxonomist forms about a taxon in a scholarly biological taxonomic publication and thus always has a taxonomic concept label. We also introduce a more general class, Operational Taxonomic Unit (:OperationalTaxonomicUnit) that can be used for all kinds of taxonomic hypotheses, including ones that don’t have a proper taxonomic concept label. The class hierarchy has been illustrated in Fig. 7.

**Fig. 8:**
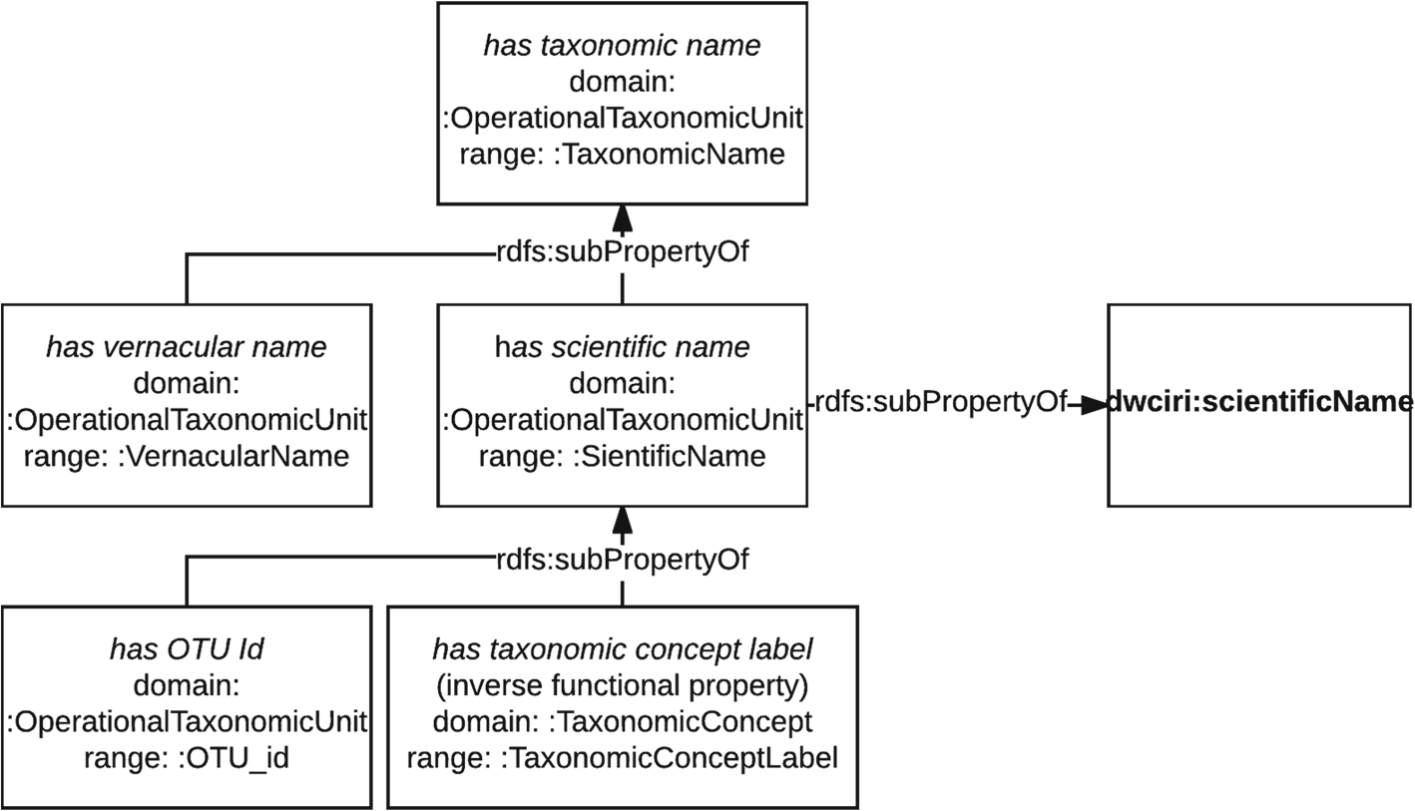
Taxonomic name property hierarchy diagram. Property hierarchy is aligned with the taxonomic name class hierarchy and with DarwinCore

Taxonomic concepts are related to taxonomic names—including taxonomic concept labels—via the property *has taxonomic name* (:taxonomicName) and its sub-properties mimicking in their range the hierarchy of taxonomic names that we introduced earlier. We have defined a property specifically to link taxonomic concepts to taxonomic concept labels, *has taxonomic concept label* (:taxonomicConceptLabel). The property hierarchy diagram is shown in Fig. 8.

**Fig. 9:**
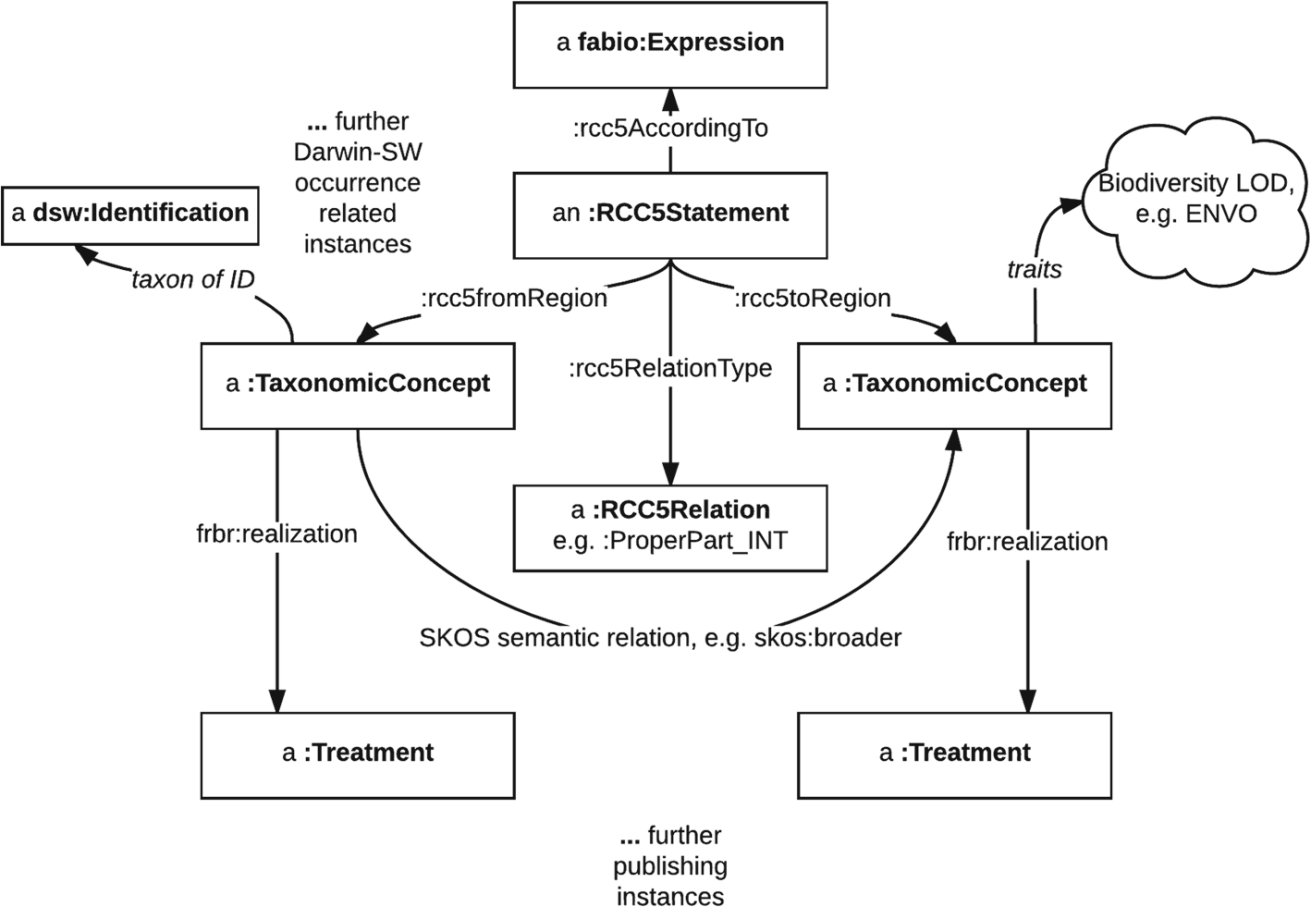
Taxonomic concept relationships diagram. In order to express an RCC-5 relationship between concepts, create an :RCC5Sgtatement and use the corresponding properties to link two taxonomic concepts via it. Further, taxonomic concepts are linked to traits (e.g. ecology in ENVO), occurrences (e.g. Darwin-SW) and realize treatments

There are two ways to relate taxonomic concepts to each other (Fig. 9). As we pointed out earlier, historically taxonomic concepts form the hierarchy known as biological taxonomy. To express such simple semantic relations, it is fully sufficient to use the SKOS semantic vocabulary [50].

However, these simple relationships are not well suited for machine reasoning. This is why Franz and Peet [30] suggested, building on previous work by e.g. [58], to use the RCC-5 language to express relationships between taxonomic concepts. Furthermore, the Euler [59] program was developed, which uses Answer Set Programming (ASP) to reason over RCC-5 taxonomic relationships. An answer set reasoner is not part of OpenBiodiv as this task can be accomplished by Euler; however, we have provided an RCC-5 dictionary class (:RCC5Dictionary), an RCC-5 relation term class (:RCC5Relation), a vocabulary of such terms to express the RCC-5 relationships in RDF (Additional file 3), as well as a class and properties to express RCC-5 statements (:RCC5Statement, :rcc5Property, and subproperties).

#### Semantics and alignment

We introduce Taxonomic Concept as equivalent (owl:equivalentClass) to the DwC term Taxon (dwc:Taxon) [60]. However, by including “concept” in the class’ name, we highlight the fact that the semantics it carries reflect the scientific theory of a given author about a taxon in nature. As we mentioned earlier, our ontology models the ongoing still unfinished process of taxonomic discovery. For this reason, we also derive Taxonomic Concept from Work. This derivation fits the definition of Work in FRBR/FaBiO, which is *“a distinct intellectual or artistic creation.”* Finally, as we use SKOS to connect taxonomic concepts to each other, we derive Taxonomic Concept from SKOS Concept.

As with other semantic publishing-related aspects of the ontology, the creation of the RCC-5 vocabulary follows the SPAR Ontologies’ model. Thus OpenBiodiv RCC-5 Vocabulary (:RCC5RelationshipTerms) is a SKOS concept scheme and every RCC-5 Relation is a SKOS concept. This allows to seamlessly share this vocabulary with other publishers of biodiversity information that also follow the SPAR Ontologies’ model.

It is important to note that we have aligned the subproperty of *has taxonomic name*, *has scientific name* (:scientificName), to the DwC property dwciri:scientificName. The difference is that while the DwC property is unbound and provides more flexibility, the OpenBiodiv-O property has the domain Taxonomic Concept and the range Scientific Name and provides for inference. Furthermore, *has taxonomic concept label* is an inverse-functional property with the domain Taxonomic Concept. This means that a given taxonomic concept label uniquely determines its taxonomic concept. This is accomplished by a minimum cardinality restriction on the property.

Together with the declaration of *has taxonomic concept label* to be an inverse functional property, we can now list what types of relationships between names and taxonomic concepts are allowed: (1) The relationship between a taxonomic concept and a name that is not a taxonomic concept label is many-to-many—i.e. one Linnaean name can be a mention of multiple taxonomic concepts, and one taxonomic concept may have multiple Linnaean names. (2) The relationship between a taxonomic concept and a taxonomic concept label is one-to-many: while a taxonomic concept may have more than one (at least one is needed) labels, every label uniquely identifies a concept. These logical restrictions make taxonomic concept labels into unique identifiers to taxonomic concepts, something that Linnaean names are not.

#### Usage

**Fig. 10:**
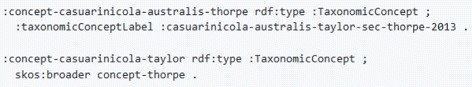
Example simple taxonomic concept relationships. We can use SKOS semantic properties to illustrate simple relationships between taxonomic concepts

**Fig. 11:**
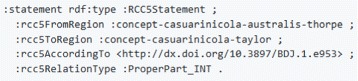
Example of RCC-5 taxonomic concept relationships. In order to express an RCC-5 relationship between concepts, create an :RCC5Sgtatement and use the corresponding properties to link two taxonomic concepts via it. SKOS relations relate concepts directly

For an example of linking two taxonomic concepts to each other, let us look at the species-rank concept *Casuarinicola australis* Taylor, 2010 sec. Thorpe [61]. It is a narrower concept than the genus-rank concept of *Casuarinicola* Taylor, 2010 sec. Taylor [62]. As we have aligned our concepts to SKOS, we can use its vocabulary to express this statement as seen in the example in Fig. 10. A further example of how to utilize the OpenBiodiv RCC-5 vocabulary is found in Fig. 11.

Furthermore, thanks to the alignment to DwC, we treat instances of our class Taxonomic Concept as functionally equivalent to DwC Taxa. This makes linking to other biodiversity ontologies possible. For example, the Open Biomedical Ontologies’ (OBO) Population and Community Ontology (PCO) [63] has a class “collection of organisms” (http://purl.obolibrary.org/obo/PCO_0000000) that can be considered a superclass of DwC Taxon. Therefore, every taxonomic concept is a collection of organisms and the application of OBO properties on it is allowed.

**Fig. 12:**
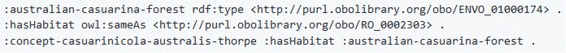
Example of combining ENVO with OpenBiodiv-O. We create a shortcut for *has habitat* and instance of the “forest biome” and link them to our taxonomic concept in order to express the fact that specimens of it have been found to live in *Casuarina* trees

In the paper that inspired our *Casuarinicola* example [61], we read: *“On 26 February 2013, the species was found to be fairly common on Casuarina trees at Thomas Bloodworth Park, Auckland.”* This statement can be interpreted (in ENVO) as meaning that the taxonomic concept that the author formulated implies that it includes the habitat “forest biome” (http://purl.obolibrary.org/obo/RO_0002303). The RDF example is shown in Fig. 12.

As we pointed out earlier, taxonomic concepts have an intensional component (traits or characters) and an ostensive component (a list of occurrences belonging to the concept). The ostensive component can be expressed by linking occurrences to the taxonomic concepts via Darwin-SW. This is possible as we have aligned the Taxon Concept class to DwC Taxon used by Darwin-SW. For an example refer to [27].

Lastly, describing traits is an active area of ontological research [64]. Due to the very complex language used to describe morphological characteristics, the Ontology Term Organizer (OTO) [64] software was developed to allow for user-created vocabularies. We will rely on such external efforts for expressing traits and trait equivalences (in the taxonomic sense) during the population of OpenBiodiv with triples. We are tightly working with the developers of OTO to integrate their efforts into OpenBiodiv [65].

**Fig. 13:**
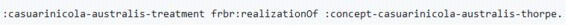
Example connection between a treatment and a taxonomic concept. A treatment is the realization of a taxonomic concept

Further, the interpretation of Taxonomic Concepts as Work means that they are realized by taxonomic treatments (e.g. Fig. 13).

## Discussion

OpenBiodiv-O is—together with the Treatment Ontologies [20]—the first effort to model taxonomic articles as RDF. It introduces classes and properties in the domains of biodiversity publishing and biological taxonomy and aligns them with the SPAR Ontologies, the Treatment Ontologies, the Open Biomedical Ontologies (OBO), TaxPub, NOMEN, and DarwinCore. We believe this introduction bridges the ontological gap that we had outlined in our aims and allows for the creation of a Linked Open Dataset (LOD) of biodiversity information (biodiversity knowledge graph [8, 66]).

Furthermore, this biodiversity knowledge graph, together with this ontology, additional semantic rules, and user software will form the OpenBiodiv Knowledge Management System. This system, as any taxonomic information system should, has taxonomic names as a key building block. For any given taxonomic name, the user will be able to rely on two patterns—*replacement name* and *related name*—to get answers to two questions of high importance to the working taxonomist. First: what is the current and historical usage of any given Linnaean name? Second: given a particular name, what other related names ought to be considered in a taxonomic discussion?

Both may be useful in building semantic search applications and the latter, in particular, is actively being researched by a group at the National Center for Text Mining in the UK (NaCTeM) [67]. OpenBiodiv-O proper does not include a mechanism for inferring replacement names and related names; however, such mechanisms are part of the OpenBiodiv knowledge system via SPARQL rules using information encoded in the document structure (Nomenclature section). Another way to infer related names is via a machine learning approach to obtain feature vectors of taxonomic names. Note that the ontology can describe related names independent of the process of their generation and will enable the comparison of both approaches in a future work.

On the other hand, by using OpenBiodiv-O, a knowledge-based system does not have to have a backbone name-based taxonomy. A backbone taxonomy is a single, monolithic hierarchy in which any and all conflicts or ambiguities have been pragmatically (socially, algorithmically) resolved, even if there is no clear consensus in the greater taxonomic domain. Such backbone taxonomies are used in systems that rely solely on taxonomic names (and not concepts) as bearers of information. They are needed as it is impossible, in such a system, to express two different sets of statements for a single name.

In OpenBiodiv, however, multiple hierarchies of taxonomic concepts may exist. For example, large synthetic taxonomies such as GBIF’s backbone taxonomy [68] or Catalogue of Life [69] may not agree or may have some issues [70]. With OpenBiodiv-O, we may, in fact, incorporate both these taxonomies at the same time! It is possible according to the ontology to have two sets of taxonomic concepts (even with the same taxonomic names) with a different hierarchical arrangement. By allowing this, we leave some room for human interpretation as an additional architectural layer. Thus, we delay the decision of which hierarchy to use to the user of the system (e.g. a practicing taxonomist) and not to the system’s architect. Due to this design feature, it is likely that our system stands a better chance to be trusted as a science process-enabling platform as the system architects don’t force a taxonomic opinion on the practicing taxonomist.

It should be noted that a successful concept-based system exists for the taxonomic order Aves (birds) [71]. The main issue that we will face is to develop tools to enable expert users to annotate taxonomic concepts with the proper relationships as only recently individual articles utilizing concept taxonomy in addition to nomenclature have been published [43, 72, 73]. We do believe that their numbers will rise driven by the realization that there are some problems with relying solely on Linnaean names for the identification of taxonomic concepts [5, 74, 75]. Concept taxonomy may, in fact, become even more important in the future as conservation efforts face challenges due to unresolved taxonomies [76]. Properly aligning taxonomic concepts to nomenclature across revisions [77] may be the solution.

Together with taxonomic information, the ontology allows modeling the source information in a knowledge base. This will be useful for metastudies, for the purposes of reproducible research, and other scholarly purposes. Moreover, it will be an expert system as the knowledge extracted will come from scholarly publications. We envision the system to be able to address a wide variety of taxonomic competency questions raised by researchers during pro-iBiosphere [78]. Examples include: “Is X a valid taxonomic name (in a nomenclatorial sense)?” “Which treatments use different names for the same taxon concepts?” “Which treatments are nomenclatorially linked (including homonyms!) to another treatment?”

Out immediate next efforts will be concentrated on populating the ontology with triples extracted from prospectively published Pensoft journals [79], legacy journals text-mined by Plazi [80], as well as databases such as GBIF and Bioimages [81]. Special effort will be made to link the dataset to the Linked Open Data cloud via resources such as geographic or institution names. In terms of extending the ontological model, more research needs to go into modeling the taxonomic concept circumscription—creating ontologies for morphological, genomic, or ecological traits. Also possibly refining the RCC-5 statements informed by the actual implementation. A study will be carried out to investigate the usefulness of the ontology once the LOD dataset had been created in a real-world scenario.

## Conclusions

The paper provides an informal conceptualization of the taxonomic process and a formalization in OpenBiodiv-O. It introduces classes and properties in the domains of biodiversity publishing and biological systematics and aligns them with the important domain-specific ontologies. By bridging the ontological gap between the publishing and the biodiversity domains, it will enable the creation of Open Biodiversity Knowledge Management System, consisting of (1) the ontology itself; (2) a Linked Open Dataset (LOD) of biodiversity information (biodiversity knowledge graph); and (3) user interface components aimed at searching, browsing and discovering knowledge in big corpora of previously dispersed scholarly publications. Through the usage of taxonomic concepts, we have included mechanisms for democratization of the scholarly process and not forcing a taxonomic opinion on the users.

## Additional files

Additional file 1 Ontology is a plain text file containing statements in the Turtle syntax forming OpenBiodiv-O. It can be edited in a text (e.g. Sublime Text, Emacs, etc.) or in an ontology editor (e.g. Protégé). It can be loaded it into a triple store (e.g. GraphDB). The prefixes that are used throughout this manuscript are defined at the beginning. This file corresponds to <http://openbiodiv.net/openbiodivo-20171103>. (TXT 22 kb)

Additional file 2 Vocabulary of Taxonomic Statuses is a plain text file containing statements in the Turtle syntax forming the OpenBiodiv Vocabulary of Taxonomic Statuses. Like the ontology [Additional file 1] it can be edited in a text or ontology editor or loaded in a triple store. Make sure you also load the ontology first. (TXT 7 kb)

Additional file 3 RCC-5 Vocabulary is a plain text file containing statements in the Turtle syntax forming the OpenBiodiv RCC-5 Vocabulary. Like the ontology [Additional file 1] it can be edited in a text or ontology editor or loaded in a triple store. Make sure you also load the ontology first. (TXT 5 kb)

## Abbreviations

LOD: Linked open data
OWL: W3C web ontology language
RCC-5: Region connection calculus 5
RDF: Resource description framework
RDFS: RDF schema
SPARQL: SPARQL protocol and RDF query language
XML: Extensible markup language
